# Pain severity and opioid use in patients with spine-related diagnoses

**DOI:** 10.3389/fpain.2025.1703201

**Published:** 2026-01-12

**Authors:** Chijioke M. Okeke, Javeria Khalid, J. Douglas Thornton, Rajender R. Aparasu

**Affiliations:** 1 The Prescription Drug Misuse Education and Research (PREMIER) Center, College of Pharmacy, University of Houston, Houston, TX, United States; 2 Department of Pharmaceutical Health Outcomes and Policy, College of Pharmacy, University of Houston, Houston, TX, United States; 3 The Center for Population Health Outcomes and Pharmacoepidemiology Education and Research (P-HOPER) Center, College of Pharmacy, University of Houston, Houston, TX, United States

**Keywords:** back pain, medical expenditure panel survey, MEPS, opioids, pain severity, spine-related diagnoses, SRD

## Abstract

**Objective:**

Clinical guidelines recommend opioid use only in severe and unresponsive cases of low back pain. Limited evidence exists on pain levels and opioid use in spine-related diagnoses (SRD). This study assessed the association between self-reported pain severity and opioid use among U.S. adults with SRD.

**Methods:**

We conducted a retrospective cross-sectional analysis using 2018–2021 Medical Expenditure Panel Survey (MEPS) data. Adults aged ≥18 with SRD diagnoses and pain prescriptions were included. Pain severity was assessed using the Veterans Rand-12 item on pain interference with routine work. Pain medication use was determined from prescription records. Multivariable logistic regression was used to evaluate the association after controlling for other factors.

**Results:**

According to the MEPS, there were 1.91 million (95% CI: 1.71–2.11) adults with SRD receiving pain medications annually, among whom 63.92% received opioid prescriptions. Most of the SRD patients were <66 years (69.71%), females (63.14%), and non-Hispanic whites (76.25%). Over half reported high pain interference with their routine activities (52.06%) Multivariable logistic regression analysis revealed that SRD patients who reported high pain interference with their routine work had higher odds of opioid use compared to those who reported low pain interference with their routine work (OR = 2.92, 95%CI: 1.49–5.70).

**Conclusion:**

Over half of SRD patients receiving pain medications reported high pain severity levels, and these high pain levels were associated with higher odds of opioid use. While this evidence aligns with the clinical recommendations, more research is needed to understand the opioid use in SRD.

## Introduction

Spine-related diagnoses (SRD) encompass a broad spectrum of musculoskeletal conditions involving the cervical, thoracic, and lumbosacral spine and represent one of the leading causes of pain and disability in the United States (1, 2). Classification of painful spinal disorders is clinically complex and typically includes conditions driven by systemic pathology (e.g., neoplasms or developmental abnormalities), those associated with neurological compromise such as nerve root compression or radiculopathy, and more common non-specific or mechanical spine pain (1, 2). Dorsalgia or back pain is reported as one of the most common reasons for emergency department visits in the United States (3) and poses a major health burden globally (4, 5). Approximately 16% of American adults report neck discomfort every year, and a proportion of these patients additionally mention having lower back pain at the same time (6). Nonpharmacologic approaches like superficial heat, massage, acupuncture, and spinal manipulation, as well as non-opioid analgesics such as nonsteroidal anti-inflammatory drugs (NSAID) or muscle relaxants, are often more recommended and are being used as first-line therapies for acute pain in this population (7, 8).

Opioid prescriptions are given to about one-fifth of outpatients who present with pain in the United States and are frequently prescribed for acute injuries (9, 10). As of 2015, about 2 million people had an opioid use disorder involving prescription opioids (11). While evidence following the implementation of the 2016 Center for Disease Control (CDC) guideline for opioid prescribing suggests that the overall outpatient prescriptions have decreased by 44%, the rate of overdose deaths due to prescription opioids still remains significant (12). In addition, opioid use for back pain strongly predicts increased length of disability, which is a significant outcome among patients with SRD (13, 14). In 2017, the American College of Physicians (ACP) published its clinical recommendations, which emphasized the avoidance of opioids in the treatment of acute low back pain (LBP) and promoting the use of NSAIDs and acetaminophen, reserving opioids for severe and unresponsive cases and for short-term use (15). Although LBP has significant health implications, limited evidence exists regarding pain levels and the extent of opioid use for LBP. Previous opioid utilization studies among the SRD population have been limited to the assessment of adverse or economic outcomes associated with opioid use (16–19), patterns of opioid use (20, 21), and racial disparities (22).

The current clinical guidelines suggest opioid use only for severe and refractory pain conditions in SRD (15). Assessing pain levels in a nationally representative sample of US population with SRD and associated opioid use will be valuable and fill the needed literature gap regarding physicians' adherence to these guidelines. Hence, this study assessed the association between self-reported pain severity and opioid utilization among patients with SRD in the United States using the Medical Expenditure Panel Survey (MEPS).

## Materials and methods

### Study design and data source

This study utilized a retrospective cross-sectional design by utilizing the 2018–2021 MEPS data (23). MEPS is a series of extensive surveys carried out by the Agency for Healthcare Research and Quality (AHRQ). It follows a panel design, consisting of five interview rounds spread over a period of two calendar years. MEPS participants are chosen using the sampling system of the National Health Interview Survey, which identifies civilian, noninstitutionalized persons residing in the United States with particular characteristics to take part in the survey. The Household Component (MEPS-HC), which is a key part of MEPS, gathers self-reported information on many aspects of household members. This includes demographic details, health conditions and status, emergency visits, inpatient stays, health insurance, income, and healthcare expenses (23). The Medical Conditions files encompass data on conditions reported by respondents, which were coded using the International Classification of Disease, 10th Edition Clinical Modification (ICD-10-CM) by professional coders and subsequently converted to clinical classification codes by the AHRQ (23). The Prescribed Medicines files are organized at the event level, with each record representing a distinct prescribed medication that includes the national drug code (NDC), medication name, and any conditions associated with the prescription procedure. NDCs were categorized into a therapeutic class according to the Multum Lexicon therapeutic classification method and provided to the researchers in the MEPS (23). MEPS data are de-identified and fully compliant with the Health Insurance Portability and Accountability Act (HIPAA); therefore, the University of Houston Institutional Review Board deemed the study exempt from human subjects' review.

### Study population

The study population included patients 18 years or older with a diagnosis of SRD and pain prescriptions. Patients were identified from the medical conditions files using the International Classification of Diseases, Tenth Edition (ICD-10-CM) codes for all SRD diagnoses (12, 16, 17). While the MEPS data includes ICD-10-CM codes, it only provides broader 3-digit categories rather than the full, detailed codes. Accordingly, we identified all specific SRD diagnoses based on their first three digits provided by MEPS. A comprehensive list of included SRD ICD-10-CM codes can be found in the Supplementary Material (S1) (12, 16, 17). Multum Lexicon therapeutic class codes were used to identify self-reported pain medication use in MEPS prescription data (19).

### Pain severity

Pain severity is a self-reported measure by MEPS participants as a part of the Veterans RAND 12-Item Health Survey (VR-12) items in the MEPS household components. This item measures the extent of pain interference with routine work. In MEPS, pain severity level is classified as extreme, high, moderate, low or no pain interference with routine work, based on responses to an item that asked MEPS respondents about their pain interference levels: “*During the past 4 weeks, how much did pain interfere with your normal work (including both work outside the home and housework)*?” (24–27). We recategorized this variable into two groups—high and low pain interference with routine work—to enhance clarity and interpretability.

### Opioid Use

Prescriptions for opioid medications were determined using the Multum Lexicon therapeutic subclassification variables of “60” (narcotic analgesics) or “191” (narcotic analgesic combinations). The prescribed opioid group consisted of patients who reported having a prescription for at least one narcotic analgesic or narcotic analgesic combination while prescriptions for non-opioid medications were extracted using the Multum Lexicon therapeutic classification variables of “61” (NSAIDS) or “73” (muscle relaxants) (28–30).

### Covariates

Covariates were identified using the conceptual framework of the Anderson Behavioral Model (ABM). According to ABM, healthcare utilization is a function of predisposing, enabling and need factors (31). *Predisposing factors* include age, gender, race/ethnicity, and region (23). *Enabling factors* include education status, income provider type, and health insurance status. *Need factors* include functional limitations, work limitations, activity of daily living (ADL) limitation, instrumental activity of daily living (IADL) limitation, perceived physical health, record of any surgical procedure, and perceived mental health status. The number of comorbidity (Elixhauser comorbidity score) was calculated as a composite score for each patient, based on the presence of 32 distinct chronic diseases, utilizing the algorithm referenced in the literature (32). Data for the included chronic diseases were obtained from the MEPS medical condition files (23). In addition, self-reported surgical procedure was included as a need variable (33, 34).

### Statistical analysis

All descriptive and multivariable analyses accounted for the complex survey design of the MEPS data and to generate nationally representative estimates. We applied the person-level full-year sampling weights provided in MEPS to adjust for differential probabilities of selection, nonresponse, and survey noncoverage. Variance estimation incorporated the MEPS stratification (VARSTR) and primary sampling unit (VARPSU) variables to account for the multistage clustered sampling design.

Descriptive weighted analyses were used to examine the characteristics of patients with SRD. Additionally, we conducted bivariate analyses using ꭓ^2^ statistics to compare the proportions of patient characteristics and opioid use. Multivariable logistic regression model was used to examine the relationship between pain severity and opioid use, accounting for all observed patient factors. All statistical analyses were conducted using SAS software 9.4 version.

## Results

### Characteristics of SRD patients receiving pain medications

**Figure 1:**
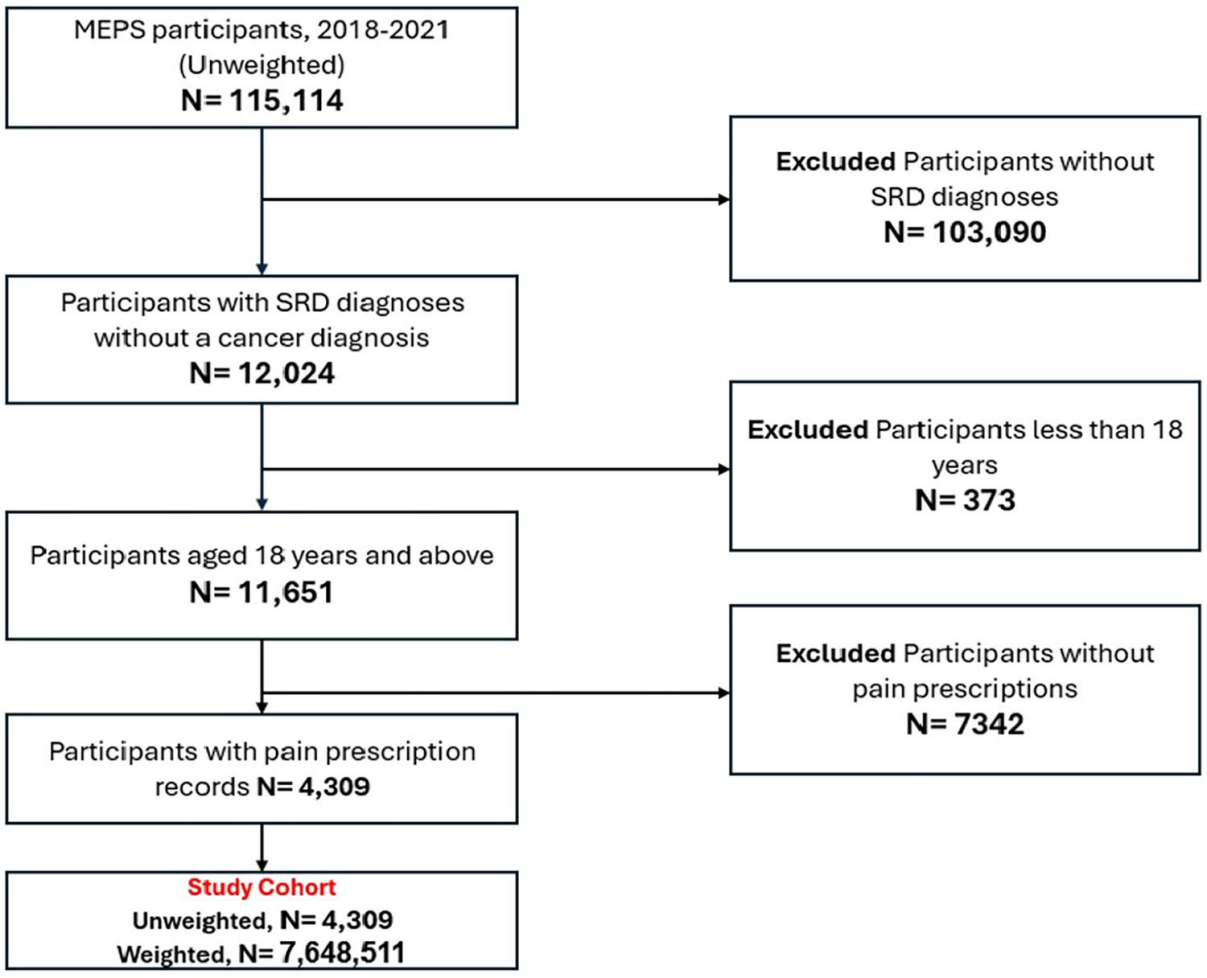
Sample selection process (MEPS, 2018–2021).

The study involved 7.64 (95% CI: 6.86–8.44) million non-institutionalized adults with SRD and pain prescriptions after applying our selection criteria (Figure 1). Approximately 64% of the participants in this study were aged between 18 and 65 years. Majority of them were females (63.14%), non-Hispanic whites (76.25%), and privately insured (55.05%). Most participants have college degrees (58.86%), were from the southern region (38.19%), and reported having high income (38.29%). Most of the respondents reported high pain interference with their routine activities (52.06%) (Table 1).

**Table 1:** Demographics and descriptive characteristics of US adult patients with spine-related diagnoses(SRD) receiving pain medications.

Variables	*N* = 7,648,511 (100%)	*N* = 4,888,722 63.92%)	*N* = 2,759,789 (36.08%)	*P*-value
	Total weighted frequency	Opioid Users	Non-opioid users	
	*N* (%)	*n* (%)	*n* (%)	
Age group, years				**0.01**
18–50	2,623,700 (34.30)	1,464,477 (29.96)	1,159,223 (42.00)	
51–65	2,708,016 (35.41)	1,774,748 (36.30)	933,269 (33.82)	
66–75	1,674,279 (21.89)	1,154,102 (23.61)	520,177 (18.85)	
75+	642,516 (8.40)	495,395 (10.13)	147,120 (5.33)	
Gender				0.18
Male	2,818,977 (36.86)	1,714,716 (35.07)	1,104,261 (40.01)	
Female	4,829,535 (63.14)	3,174,006 (64.93)	1,655,529 (59.99)	
Race/Ethnicity				0.25
Hispanic	697,193 (9.12)	364,081 (7.45)	333,112 (12.07)	
Non-Hispanic black	701,733 (9.17)	437,065 (8.94)	264,668 (9.59)	
Non-Hispanic others	417,583 (5.46)	269,193 (5.51)	148,390 (5.38)	
Non-Hispanic whites	5,832,003 (76.25)	3,818,384 (78.10)	2,013,619 (72.96)	
Insurance type				0.28
Private	4,210,243 (55.05)	2,662,600 (54.46)	1,547,643 (56.08)	
Public	3,302,371 (43.18)	2,165,061 (44.29)	1,137,310 (41.21)	
Uninsured	135,897 (1.78)	61,061 (1.25)	74,836 (2.71)	
Region				**0.0018**
Northeast	1,063,367 (13.90)	518,384 (10.60)	544,984 (19.75)	
Midwest	1,784,716 (23.33)	1,174,339 (24.02)	610,377 (22.12)	
South	2,921,275 (38.19)	1,829,821 (37.43)	10,91,454 (39.55)	
West	1,879,152 (24.57)	1,366,177 (27.95)	512,975 (18.59)	
Education level^a^				**0.003**
College or more	4,502,127 (58.86)	2,725,531 (55.75)	1,776,596 (64.37)	
High school or less	3,070,366 (40.14)	2,091,775 (42.79)	978,591 (35.46)	
Income				0.92
Poor/Negative	1,272,802 (16.64)	784,592 (16.05)	488,210 (17.69)	
Near Poor	351,790 (4.60)	222,579 (4.55)	129,211 (4.68)	
Low Income	1,019,195 (13.33)	696,625 (14.25)	322,571 (11.69)	
Middle Income	2,076,416 (27.15)	1,310,748 (26.81)	765,668 (27.74)	
High Income	2,928,308 (38.29)	1,874,178 (38.34)	1,054,130 (38.20)	
Provider Type^a^				0.25
Facility	1,310,395 (17.13)	822,070 (16.82)	488,324 (17.69)	
Person	1,290,397 (16.87)	848,147 (17.35)	442,250 (16.02)	
Person-in-facility	4,205,242 (54.98)	2,770,680 (56.67)	1,434,562 (51.98)	
Had any Surgical Procedure				**<.0001**
No	4,742,076 (62.00)	2,994,342 (61.25)	22,13,074 (80.19)	
Yes	2,906,435 (38.00)	1,894,380 (38.75)	546,715 (19.81)	
Year				0.53
2018	3,171,849 (41.47)	2,071,046 (42.36)	1,100,803 (39.89)	
2019	2,018,147 (26.39)	1,266,287 (25.90)	751,860 (27.24)	
2020	1,238,165 (16.19)	806,021 (16.49)	432,144 (15.66)	
2021	1,220,350 (15.96)	745,368 (15.25)	474,982 (17.21)	
Activity of daily living (ADL) Limitation^a^				0.72
No	7,136,404 (93.30)	4,547,571 (93.02)	25,88,833 (93.81)	
Yes	499,061 (6.52)	337,073 (6.89)	161,988 (5.87)	
Instrumental Activity of Daily Living (IADL) Limitation^a^				
No	6,821,397 (89.19)	4,266,590 (87.27)	25,54,807 (92.57)	0.12
Yes	808,332 (10.57)	612,317 (12.53)	196,015 (7.10)	
Functional Limitation^a^				**0.02**
No	3,866,893 (50.56)	2,283,311 (46.71)	15,83,582 (57.38)	
Yes	3,761,925 (49.19)	2,594,686 (53.07)	11,67,239 (42.29)	
Work Limitation^a^				**0.03**
No	4,717,410 (61.68)	2,824,396 (57.77)	18,93,014 (68.59)	
Yes	2,903,947 (37.97)	2,046,139 (41.85)	857,808 (31.08)	
Number of Comorbidities^a^				0.07
0	1,220,469 (15.96)	618,932 (12.66)	601,537 (21.80)	
1	1,446,012 (18.91)	917,825 (18.77)	528,186 (19.14)	
2	1,159,184 (15.16)	788,745 (16.13)	370,439 (13.42)	
3	1,238,058 (16.19)	856,687 (17.52)	381,372 (13.82)	
4	845,802 (11.06)	600,979 (12.29)	244,822 (8.87)	
5+	1,738,987 (22.74)	1,105,553 (22.61)	633,434 (22.95)	
Pain severity^a^				**<.0001**
High pain interference	3,981,571 (52.06)	2,856,586 (58.43)	11,24,985 (40.76)	
Low pain interference	3,048,452 (39.86)	1,626,388 (33.27)	14,22,064 (51.53)	

a Variable contains missing values, % may not add up to 100%.

**Bolded *P*-values**: significant *p*-values.

### Opioid utilization by patients with SRD

Approximately 5 million patients with SRD (63.92%; 95% CI: 60.05%–67.78%) received an opioid prescription (Table 1). Among opioid users, 2.86 million (58.43%) reported high pain severity. In contrast, among the 2.76 million patients who did not receive opioids, 40.76% reported high pain levels (*P* < 0.0001) (Table 1).

### Multivariable analyses

Table 2 shows the adjusted association between pain severity levels and opioid use among SRD patients after accounting for patient characteristics. We found that SRD patients with pain prescriptions who reported high pain interference with their routine work had higher odds of opioid use compared to those who reported low pain interference with their routine work (OR = 2.92, 95%CI: 1.49–5.70). Some other significant factors associated with opioid use were age (18–50 years vs. ≥75years: OR = 0.30, 95%CI: 0.10–0.87), gender (Male vs. Female: OR = 0.27, 95%CI: 0.15–0.49), region (Northeast vs. West: OR = 0.23, 95%CI: 0.11–0.48; South vs. West: OR=0.42, 95%CI: 0.23–0.78), educational level (College or more vs. High school or less: OR = 0.50, 95%CI: 0.31–0.82), and surgical procedure (Yes vs. No: OR = 3.71, 95%CI: 2.18–6.31).

**Table 2:** Adjusted estimates of the association between pain severity level, other characteristics and opioid use.

Variables	Opioid Use
	OR (95% CI)
**Pain severity**	
High pain interference	**2.92 (1.49–5.70)**
Low pain interference	Reference
Age group, years	
18–50	**0.30 (0.10–0.87)**
51–65	0.36 (0.13–0.98)
66–75	0.42 (0.15–1.17)
75+	Reference
Gender	
Male	**0.27 (0.15–0.49)**
Female	Reference
Race/Ethnicity	
Hispanic	0.61 (0.30–1.26)
Non-Hispanic black	1.59 (0.78–3.24)
Non-Hispanic others	1.48 (0.60–3.39)
Non-Hispanic whites	Reference
Insurance type	
Public	1.41 (0.80–2.49)
Uninsured	0.87 (0.22–3.42)
Private	Reference
Region	
Northeast	**0.23 (0.11–0.48)**
Midwest	0.72 (0.36–1.44)
South	**0.42 (0.23–0.78)**
West	Reference
Education level	
College or more	**0.50 (0.31–0.82)**
High school or less	Reference
Income	
Poor/Negative	0.88 (0.42–1.86)
Near Poor	0.63 (0.23–1.72)
Low Income	1.28 (0.60–2.71)
Middle Income	1.04 (0.59–1.83)
High Income	Reference
Provider type	
Facility	1.49 (0.76–2.91)
Person	1.22 (0.66–2.29)
Person-in-facility	Reference
Activity of Daily Living (ADL) Limitation	
No	Reference
Yes	4.87 (0.62–3.85)
Instrumental Activity of Daily Living (IADL) Limitation	
No	Reference
Yes	1.40 (0.62–3.18)
Functional Limitation	
No	Reference
Yes	1.19 (0.66–2.13)
Work Limitation	
No	Reference
Yes	0.83 (0.45–1.53)
Had any Surgical Procedure	
No	Reference
Yes	**3.71 (2.18–6.31)**
Number of comorbidities	
0	Reference
1	**2.73 (1.29–5.79)**
2	**2.46 (1.15–5.26)**
3	**3.32 (1.41–7.86)**
4	0.54 (0.27–1.09)
5+	2.02 (0.88–4.61)
Year	
2018	2.14 (1.20–3.84)
2019	1.58 (0.93–2.68)
2020	**2.11 (1.19–3.74)**
2021	Reference

**OR:** Odds ratio.

**Bolded OR**: significant *p*-values.

**95% CI:** 95% confidence interval.

## Discussion

We evaluated pain severity and opioid utilization among non-institutionalized US adults with SRD and pain prescriptions. Notably, one in two patients included in our study reported high pain severity levels. This is consistent with previous reports in the literature which suggest that SRD patients typically experience high pain levels often resulting in activity limitations or work-related disabilities (4, 35–38). A MEPS study found that more than half of the respondents reported limiting low back pain (38). Our estimate is however higher than a previously reported national estimate of pain severity among all adults in the US. Nahin et al. found that just over 10% of U.S. adults reported experiencing “a lot of pain” (39). The higher prevalence observed in our study may be attributed to the specific characteristics of our study population. Unlike the general adult population assessed in Nahin's study, our analysis focused exclusively on patients with SRD who also had records of pain medication prescriptions. This subset likely represents individuals with more severe or persistent pain requiring pharmacologic management, whereas the previous study captured a broader and more heterogeneous population that included individuals with varying pain severities, including those without pain. Pain is a hallmark symptom of SRDs, frequently resulting from structural abnormalities that compress the spinal cord or adjacent nerve roots, thereby limiting mobility and functional capacity (40, 41). This constitutes a significant public health concern and leads to high healthcare utilization and consequently high healthcare expenditures (42, 43).

We also found that roughly two in three SRD patients received an opioid prescription. This high rate of opioid use among SRD patients aligns with prior research that shows individuals with back and neck pain are among the most frequent recipients of opioid therapy in the U.S (9, 11, 44). Daubresse et al. utilized national prescription data and found that musculoskeletal conditions, including back pain, accounted for a substantial proportion of opioid prescriptions among adults (9). Deyo et al. also found that approximately 40%–50% of patients with chronic back pain were prescribed opioids in outpatient settings (44). Our findings support these studies and highlight the current high opioid utilization rates in SRD.

Our principal finding revealed that adult SRD patients who reported high pain interference were over two times more likely to receive opioid prescriptions compared to those who reported low pain interference with their routine work. These findings align with clinical recommendations from the ACP, which emphasize reserving opioids for severe cases of back pain (15). These findings also corroborate with a recent study in England, which showed that opioid utilization was significantly and positively associated with pain intensity (45). Although their analyses were limited to chronic pain intensity, ours was not specific to the chronicity of pain intensity but presented a general assessment of pain levels among these patients, regardless of duration. SRD patients can have diverse pain experiences, including acute post-operative pain, pre-operative and peri-operative pain, all of which can impact the potential of being prescribed opioids. Our study, therefore, is more general and represents a vital step in characterizing the correlation of pain levels and opioid use in the overall SRD patients receiving pain-related medications.

Given that multiple randomized controlled trials and observational studies have linked long-term opioid use to adverse outcomes (46–51), including functional disability, greater efforts are needed to reduce inappropriate initial prescribing. This analysis is particularly timely and relevant, as back pain remains one of the leading causes of outpatient visits for pain management in the United States. The evidence presented in this study contributes to ongoing efforts to understand opioid use and minimize associated harms. The study findings can inform policy and clinical practice for patients with SRDs and broader populations of non-cancer opioid users.

Our findings should be interpreted in the context of the following limitations. First, the implications of our findings may be constrained by insufficient data, such as clinical characteristics and prescriber-level information that may be associated with both pain and opioid use. Despite controlling for numerous other available patient factors, our analyses may be liable to residual confounding. Also, our comparator group included other non-opioid pharmacotherapy, and this may have influenced the findings, as it is limited to those needing prescription pain medications. In addition, using secondary data cross-sectionally does not allow for the determination of a cause-and-effect relationship. Future research should consider using longitudinal designs to more robustly assess this association, especially opioid intensity and long-term use. MEPS utilizes self-reported data, which may be susceptible to recall bias. Regardless of all of these limitations, it is important to note that our study provides important insights that fill the evidence gap in pain management in SRD patients.

## Conclusion

This national study found that more than half of SRD patients receiving pain medications reported high pain severity levels, and these high pain levels were associated with higher odds of opioid use compared to those with lower pain severity levels. While this evidence aligns with pain management clinical recommendations, the study findings contribute to the evidence gap and provide insights regarding the pain and the need for cautious prescribing of pain medications among SRD patients. More research is needed to understand this relationship within the context of the duration of pain and opioid intensity to provide guidance to support physicians' efforts and strategies in managing pain while optimizing opioid use among SRD patients.

## Data Availability

The datasets used and/or analyzed during the current study are publicly available on the Agency for Healthcare Research and Quality (AHRQ) website - https://meps.ahrq.gov/mepsweb/.
