# Supplementary material for: Pain severity and opioid use in patients with spine-related diagnoses
Source: Front Pain Res (Lausanne). 2026 Jan 12;6:1703201. doi: 10.3389/fpain.2025.1703201 (PMC12833261; doi:10.3389/fpain.2025.1703201)
Supplement: Supplementary file 1 [file Table1.docx]

**S1:** Diagnoses codes for spine-related disorders

| ICD-10 Descriptor | ICD-10 Code |
| --- | --- |
| Cervical |  |
| Cervicalgia | M54.2 |
| Other cervical disc degeneration,  unspecified cervical region | M50.30 |
| Other cervical disc displacement,  unspecified cervical region | M50.20 |
| Cervical disc disorder with myelopathy, unspecified cervical  region | M50.00 |
| Other cervical disc disorders, unspecified cervical region | M50.80 |
| Cervical disc disorder, unspecified, unspecified cervical region | M50.90 |
| Dislocation of unspecified cervical  vertebrae, initial encounter | S13.101A |
| Fracture of neck, unspecified, initial  encounter | S12.9XXA |
| Collapsed vertebra, not elsewhere classified, site unspecified, initial  encounter for fracture | M48.50XA |
| Age-related osteoporosis with current  pathological fracture, vertebra(e), initial encounter for fracture | M80.08XA |
| Pathological fracture, other site,  initial encounter for fracture | M84.48XA |
| Pathological fracture in other disease,  other site, initial encounter for fracture | M84.68XA |
| Unspecified displaced fracture of first  cervical vertebra, initial encounter for closed fracture | S12.000A |
| Unspecified nondisplaced fracture of | S12.001A |

| first cervical vertebra, initial  encounter for closed fracture |  |
| --- | --- |
| Unspecified displaced fracture of  second cervical vertebra, initial encounter for closed fracture | S12.100A |
| Unspecified nondisplaced fracture of second cervical vertebra, initial  encounter for closed fracture | S12.101A |
| Unspecified displaced fracture of third cervical vertebra, initial  encounter for closed fracture | S12.200A |
| Unspecified nondisplaced fracture of  third cervical vertebra, initial encounter for closed fracture | S12.201A |
| Unspecified displaced fracture of fourth cervical vertebra, initial  encounter for closed fracture | S12.300A |
| Unspecified nondisplaced fracture of fourth cervical vertebra, initial  encounter for closed fracture | S12.301A |
| Unspecified injury at C1 level of  cervical spinal cord, initial encounter | S14.101A |
| Unspecified injury at C2 level of  cervical spinal cord, initial encounter | S14.102A |
| Unspecified injury at C3 level of  cervical spinal cord, initial encounter | S14.103A |
| Unspecified injury at C4 level of  cervical spinal cord, initial encounter | S14.104A |
| Unspecified displaced fracture of fifth  cervical vertebra, initial encounter for closed fracture | S12.400A |
| Unspecified nondisplaced fracture of fifth cervical vertebra, initial  encounter for closed fracture | S12.401A |
| Unspecified displaced fracture of sixth cervical vertebra, initial encounter for  closed fracture | S12.500A |
| Unspecified nondisplaced fracture of sixth cervical vertebra, initial  encounter for closed fracture | S12.501A |
| Unspecified displaced fracture of seventh cervical vertebra, initial  encounter for closed fracture | S12.600A |
| Unspecified nondisplaced fracture of  seventh cervical vertebra, initial encounter for closed fracture | S12.601A |
| Unspecified injury at C5 level of  cervical spinal cord, initial encounter | S14.105A |
| Unspecified injury at C6 level of  cervical spinal cord, initial encounter | S14.106A |
| Unspecified injury at C7 level of  cervical spinal cord, initial encounter | S14.107A |

| Osteomyelitis of vertebra, site  unspecified | M46.20 |
| --- | --- |
| Other specific joint derangements of  unspecified joint, not elsewhere classified | M24.80 |
| Postlaminectomy syndrome, not  elsewhere classified | M96.1 |
| Radiculopathy, cervical region | M54.12 |
| Radiculopathy, cervicothoracic region | M54.13 |
| Spinal stenosis, cervical region | M48.02 |
| Spondylosis without myelopathy or  radiculopathy, cervical region | M47.812 |
| Other spondylosis with myelopathy,  cervical region | M47.12 |
| Sprain of ligaments of cervical spine,  initial encounter | S13.4XXA |
| Sprain of joints and ligaments of other  parts of neck, initial encounter | S13.8XXA |
|  |  |
| ***Thoracic*** |  |
| Radiculopathy, thoracic region | M54.14 |
| Radiculopathy, thoracolumbar region | M54.15 |
| Radiculopathy, lumbar region | M54.16 |
| Radiculopathy, lumbosacral region | M54.17 |
| Pain in thoracic spine | M54.6 |
| Chondrocostal junction syndrome  [Tietze] | M94.0 |
| Other intervertebral disc  degeneration, thoracic region | M51.34 |
| Other intervertebral disc  degeneration, thoracolumbar region | M51.35 |
| Other intervertebral disc  displacement, thoracic region | M51.24 |
| Other intervertebral disc  displacement, thoracolumbar region | M51.25 |
| Intervertebral disc disorders with  myelopathy, thoracic region | M51.04 |
| Intervertebral disc disorders with  myelopathy, thoracolumbar region | M51.05 |
| Discitis, unspecified, thoracolumbar  region | M46.45 |
| Other intervertebral disc disorders,  thoracic region | M51.84 |
| Other intervertebral disc disorders,  thoracolumbar region | M51.85 |
| Spondylopathy, unspecified | M48.9 |
| Dislocation of unspecified thoracic  vertebra, initial encounter | S23.101A |
| Unspecified fracture of unspecified  thoracic vertebra, initial encounter for closed fracture | S22.009A |

| Collapsed vertebra, not elsewhere  classified, site unspecified, initial encounter for fracture | M48.50XA |
| --- | --- |
| Age-related osteoporosis with current pathological fracture, vertebra(e),  initial encounter for fracture | M80.08XA |
| Pathological fracture, other site,  initial encounter for fracture | M84.48XA |
| Pathological fracture in other disease, other site, initial encounter for  fracture | M84.68XA |
| Unspecified fracture of first thoracic  vertebra, initial encounter for closed fracture | S22.019A |
| Unspecified fracture of second thoracic vertebra, initial encounter  for closed fracture | S22.029A |
| Unspecified fracture of third thoracic vertebra, initial encounter for closed  fracture | S22.039A |
| Unspecified fracture of fourth  thoracic vertebra, initial encounter for closed fracture | S22.049A |
| Unspecified fracture of T5-T6 vertebra, initial encounter for closed  fracture | S22.059A |
| Unspecified injury at T1 level of  thoracic spinal cord, initial encounter | S24.101A |
| Unspecified injury at T2-T6 level of  thoracic spinal cord, initial encounter | S24.102A |
| Unspecified fracture of T7-T8  vertebra, initial encounter for closed fracture | S22.069A |
| Unspecified fracture of T9-T10 vertebra, initial encounter for closed  fracture | S22.079A |
| Unspecified fracture of T11-T12  vertebra, initial encounter for closed fracture | S22.089A |
| Unspecified injury at T7-T10 level of  thoracic spinal cord, initial encounter | S24.103A |
| Unspecified injury at T11-T12 level of  thoracic spinal cord, initial encounter | S24.104A |
| Postural kyphosis, site unspecified | M40.00 |
| Unspecified kyphosis, site unspecified | M40.209 |
| Juvenile osteochondrosis of spine,  site unspecified | M42.00 |
| Other kyphosis, site unspecified | M40.299 |
| Discitis, unspecified, thoracolumbar  region | M46.45 |
| Other intervertebral disc disorders,  thoracic region | M51.84 |

| Other intervertebral disc disorders,  thoracolumbar region | M51.85 |
| --- | --- |
| Osteomyelitis of vertebra, site  unspecified | M46.20 |
| Ankylosing spondylitis of unspecified  sites in spine | M45.9 |
| Postlaminectomy syndrome, not  elsewhere classified | M96.1 |
| Other idiopathic scoliosis, site  unspecified | M41.20 |
| Neuromuscular scoliosis, site  unspecified | M41.40 |
| Other secondary scoliosis, site  unspecified | M41.50 |
| Spinal stenosis, thoracic region | M48.04 |
| Spondylosis without myelopathy or  radiculopathy, thoracic region | M47.814 |
| Other spondylosis with myelopathy,  thoracic region | M47.14 |
| Sprain of ligaments of thoracic spine,  initial encounter | S23.3XXA |
| Sprain of other unspecified parts of  thorax, initial encounter | S23.8XXA |
| Sprain of other parts of lumbar spine  and pelvis, initial encounter | S33.8XXA |
|  |  |
| Miscellaneous |  |
| Contusion of lower back and pelvis,  initial encounter | S30.0XXA |
|  |  |
| Lumbar |  |
| Low back pain | M54.5 |
| Sacrococcygeal disorders, not  elsewhere classified | M53.3 |
| Other intervertebral disc  degeneration, lumbar region | M51.36 |
| Other intervertebral disc  degeneration, lumbosacral region | M51.37 |
| Other intervertebral disc  displacement, lumbar region | M51.26 |
| Other intervertebral disc  displacement, lumbosacral region | M51.27 |
| Intervertebral disc disorders with  myelopathy, lumbar region | M51.06 |
| Intervertebral disc disorders with  myelopathy, lumbosacral region | M51.07 |
| Discitis, unspecified, lumbosacral  region | M46.47 |
| Other intervertebral disc disorders,  lumbar region | M51.86 |
| Other intervertebral disc disorders, | M51.87 |

| lumbosacral region |  |
| --- | --- |
| Unspecified fracture of unspecified lumbar vertebra, initial encounter for  closed fracture | S32.009A |
| Unspecified fracture of sacrum, initial  encounter for closed fracture | S32.10XA |
| Fracture of coccyx, initial encounter  for closed fracture | S32.2XXA |
| Unspecified fracture of unspecified lumbar vertebra, initial encounter for  closed fracture | S32.009A |
| Unspecified fracture of first lumbar  vertebra, initial encounter for closed fracture | S32.019A |
| Unspecified fracture of second lumbar vertebra, initial encounter for  closed fracture | S32.029A |
| Unspecified fracture of third lumbar vertebra, initial encounter for closed  fracture | S32.039A |
| Unspecified fracture of fourth lumbar  vertebra, initial encounter for closed fracture | S32.049A |
| Unspecified fracture of fifth lumbar vertebra, initial encounter for closed  fracture | S32.059A |
| Unspecified injury to L1 level of  lumbar spinal cord, initial encounter | S34.101A |
| Unspecified injury to L2 level of  lumbar spinal cord, initial encounter | S34.102A |
| Unspecified injury to L3 level of  lumbar spinal cord, initial encounter | S34.103A |
| Unspecified injury to L4 level of  lumbar spinal cord, initial encounter | S34.104A |
| Unspecified injury to L5 level of  lumbar spinal cord, initial encounter | S34.105A |
| Unspecified injury to unspecified level  of lumbar spinal cord, initial encounter | S34.109A |
| Complete lesion of L1 level of lumbar  spinal cord, initial encounter | S34.111A |
| Complete lesion of L2 level of lumbar  spinal cord, initial encounter | S34.112A |
| Complete lesion of L3 level of lumbar  spinal cord, initial encounter | S34.113A |
| Complete lesion of L4 level of lumbar  spinal cord, initial encounter | S34.114A |
| Complete lesion of L5 level of lumbar  spinal cord, initial encounter | S34.115A |
| Complete lesion of unspecified level of lumbar spinal cord, initial  encounter | S34.119A |

| Incomplete lesion of L1 level of  lumbar spinal cord, initial encounter | S34.121A |
| --- | --- |
| Incomplete lesion of L2 level of  lumbar spinal cord, initial encounter | S34.122A |
| Incomplete lesion of L3 level of  lumbar spinal cord, initial encounter | S34.123A |
| Incomplete lesion of L4 level of  lumbar spinal cord, initial encounter | S34.124A |
| Incomplete lesion of L5 level of  lumbar spinal cord, initial encounter | S34.125A |
| Incomplete lesion of unspecified level of lumbar spinal cord, initial  encounter | S34.129A |
| Collapsed vertebra, not elsewhere classified, site unspecified, initial  encounter for fracture | M48.50XA |
| Age-related osteoporosis with current  pathological fracture, vertebra(e), initial encounter for fracture | M80.08XA |
| Pathological fracture, other site,  initial encounter for fracture | M84.48XA |
| Pathological fracture in other disease, other site, initial encounter for  fracture | M84.68XA |
| Osteomyelitis of vertebra, site  unspecified | M46.20 |
| Postlaminectomy syndrome, not  elsewhere classified | M96.1 |
| Radiculopathy, thoracic region | M54.14 |
| Radiculopathy, thoracolumbar region | M54.15 |
| Radiculopathy, lumbar region | M54.16 |
| Radiculopathy, lumbosacral region | M54.17 |
| Sciatica, unspecified site | M54.30 |
| Spinal stenosis, lumbar region | M48.06 |
| Spondylosis, site unspecified | M43.00 |
| Spondylolisthesis, site unspecified | M43.10 |
| Congenital spondylolisthesis | Q76.2 |
| Spondylosis without myelopathy or  radiculopathy, lumbosacral region | M47.817 |
| Other spondylosis with myelopathy,  lumbar region | M47.16 |
| Sprain of ligaments of lumbar spine,  initial encounter | S33.5XXA |

**Notes:** While the MEPS data includes ICD-10-CM codes, it only provides broader 3-digit categories rather than the full, detailed codes. Accordingly, we identified all specific SRD diagnoses based on their first three digits provided by MEPS.
